# Prebiotic Effects of Insoluble Konjac Glucomannan Derived from Edible “Konnyaku” on Weight Control

**DOI:** 10.3390/microorganisms13040877

**Published:** 2025-04-11

**Authors:** Chikako Shimokawa, Wakana Mizutani, Haruhisa Motegi, Naomi Gokan, Junichi Tomita, Hajime Hisaeda

**Affiliations:** 1 Department of Parasitology, National Institute of Infectious Diseases, 1-23-1 Toyama, Shinjuku 162-8640, Tokyo, Japan; chikakos@niid.go.jp; 2 Department of Parasitology, Graduate School of Medical Science, Gunma University, 3-39-15 Showa, Maebashi 371-8511, Gunma, Japan; mizuwa@gunma-u.ac.jp; 3 ORIHIRO Plantdew Co., Ltd., 613 Shimooshima-machi, Takasaki 370-0886, Gunma, Japan; motegi-haruhisa@orihiro.com (H.M.); gokan@orihiro.com (N.G.); tomita@orihiro.com (J.T.)

**Keywords:** obesity, insoluble glucomannan, konnyaku, intestinal microbiota, leptin

## Abstract

Obesity is a major global health issue, and novel dietary approaches are needed for prevention and management. This study investigates the effect of insoluble konjac glucomannan (iKGM) derived from edible konnyaku, a traditional Japanese food, on weight gain suppression in mice. Mice treated with iKGM showed increased fecal volume, reduced food intake, and suppressed weight gain (Day 21; *p* < 0.01). This weight-suppression effect was prebiotic rather than physical properties of iKGM, as antibiotic treatment abolished the weight-suppressing effect despite increased fecal volume. iKGM treatment altered the gut microbiota, notably increasing *Akkermansia muciniphila* (Day 21; *p* < 0.01), a bacterium associated with weight loss, along with elevated levels of short-chain fatty acids (SCFAs) such as butyrate and propionate (Day 21; *p* < 0.01). Furthermore, iKGM-induced weight suppression was linked to elevated leptin levels (Day 21; *p* < 0.01), an appetite suppressant induced by SCFAs. These results suggest that iKGM modulates gut microbiota, increases *A. muciniphila*, induces leptin production, and reduces food intake, inhibiting weight gain. This study indicates that iKGM may represent a promising approach for obesity prevention through gut microbiota modulation. Future research should investigate the mechanisms of iKGM’s effects on microbiota and explore its long-term safety and efficacy in clinical trials.

## 1. Introduction

The global rise in obesity, exacerbated by industrialization and urbanization, has become a major public health crisis, impacting millions worldwide [1]. This complex condition is influenced by a range of factors, including genetics, environmental influences, and unhealthy lifestyle habits such as poor diet and physical inactivity [2]. Obesity is a leading risk factor for numerous chronic diseases, including dyslipidemia, hypertension, and arteriosclerosis, significantly contributing to the global burden of non-communicable diseases [3,4]. Additionally, the economic burden of obesity-related diseases is overwhelming, with substantial healthcare costs, reducing productivity, and contributing to socio-economic inequalities [5,6]. Given these challenges, it is crucial to explore novel and sustainable approaches to both the prevention and management of obesity.

Konnyaku potatoes (*Amorphophallus konjac*) are widely cultivated as a food source in Asian countries. Konnyaku, derived from potatoes, is a traditional Japanese food known for its high dietary fiber content and health benefits. The main components of konjac flour (KF) refined from konnyaku potatoes are water-soluble dietary fiber and glucomannan, a polysaccharide consisting of glucose and mannose through a β-1,4 bond [7]. When KF is dissolved in water and a coagulant such as calcium hydroxide is added to solidify it, it becomes ‘konnyaku’, a gel-like food that is approximately 97% water and rich in essential minerals, like calcium and magnesium. During this solidification process, hydrophilic acetyl residues in the water-soluble konjac glucomannan (KGM) are removed, resulting in insoluble KGM (iKGM) [8]. This transformation allows iKGM to be metabolized in the gut, where it interacts with gut microbiota to promote the production of short-chain fatty acids (SCFAs). SCFAs are implicated in metabolic regulation, including appetite control and energy metabolism, suggesting a role for konnyaku in weight management and metabolic health. Additionally, KGM expands in the stomach, promoting satiety and potentially reducing overall caloric intake. With its low-calorie, high-fiber composition and beneficial effects on gut microbiota, konnyaku is increasingly recognized as a functional food with potential applications in obesity and metabolic disease research.

The human gut harbors a complex microbial community, comprising up to 100 trillion bacteria from over 1000 different species, collectively known as the gut microbiota [9]. These microbes play an essential role in maintaining homeostasis and influencing the pathogenesis of various diseases, including obesity [10,11]. Notably, certain gut bacteria have been linked to obesity, with *Akkermansia muciniphila* (*A. muciniphila*) being identified as a “lean bacterium” that may help prevent weight gain [12,13]. A growing body of evidence suggests that the gut microbiota exerts its effects on energy metabolism through the production of metabolites like short-chain fatty acids (SCFAs), including butyrate and propionate, which influence various physiological processes, including appetite regulation and energy balance [14,15,16]. SCFAs, in particular, have been shown to suppress eating behavior by stimulating leptin secretion from adipocytes, a key regulator of appetite and energy expenditure [17,18]. Given that dietary fibers, such as KGM, are not digestible by human enzymes but are instead fermented by gut bacteria to produce SCFAs, they are thought to play a crucial role in regulating metabolism through prebiotic mechanisms [19,20].

Soluble KGM has been shown to modulate gut microbiota composition, reduce blood lipids, and improve immune function [21,22,23]. However, the impact of iKGM, the insoluble form of KGM, on metabolism, obesity, and gut microbiota remains largely underexplored. This study investigates the effects of iKGM from konnyaku on weight gain and its potential prebiotic effects using a mouse model. Our findings reveal that iKGM significantly alters the gut microbiota, leading to a reduction in weight gain. We demonstrate that these effects are associated with increased leptin levels and changes in intestinal bacteria, specifically *Akkermansia muciniphila*, providing new insights into the potential of iKGM as a therapeutic agent for obesity management.

## 2. Materials and Methods

### 2.1. Animal Model and Study Design

Eight-week-old female C57BL/6J mice, procured from Japan SLC (Hamamatsu, Japan), were housed under specific pathogen-free conditions. Experiments were conducted when the mice reached 10 to 12 weeks of age. The study was reviewed and authorized by the Animal Ethics Committee of the Graduate School of Gunma University (approval number 16-041; approval date: 20 September 2016) and was performed in compliance with the institutional guidelines for animal experimentation, as well as Japanese government regulations (law No. 105 and notification No. 6). The mice were randomly assigned to either a control group or an iKGM-treated group. The iKGM was orally administered at a dosage of 120 mg/kg/day, which corresponds to a human equivalent of 7.2 g/day for a 60 kg individual. The mean initial body weight of the mice was 22.5 g, and the daily dose per mouse was calculated as follows: 120 mg/kg × 0.025 kg = 2.7 mg. Throughout the experiment, food and water consumption were monitored, and fecal and urinary output was measured. Serum, intestinal contents, and tissue samples were periodically collected for analysis. The feed efficiency ratio (FER) was computed as weight gain (g) per gram of food intake, while the feed conversion ratio (FCR) was defined as food consumption (g) per gram of body weight gain.

### 2.2. Preparation of Insoluble Konjac Glucomannan (iKGM)

Konjac flour, derived from the tubers of *Amorphophallus konjac*, was first dissolved in warm water to create a thick, gelatinous paste. To initiate the coagulation process, calcium hydroxide (Ca(OH)_2_) was added to the paste. The resulting mixture was carefully heated in hot water at a controlled temperature range of 80–90 °C for 100–120 min. This heating process not only facilitated the formation of a solidified konnyaku gel but also allowed for the partial removal of soluble components such as acetyl groups from the glucomannan polysaccharide. The konnyaku gel was then cut into small chunks and subjected to mechanical grinding to break it down into smaller, more uniform particles. Following this, the ground konnyaku was dehydrated using a controlled drying process. Initially, the grated konnyaku pieces were soaked in water to rehydrate and soften the material, ensuring that the texture was appropriate for the next stage. After soaking, the konnyaku was dried under low heat, transforming it into thin, flexible sheets of dried konjac film. These dried films were then finely milled into a powder, resulting in the preparation of insoluble konjac glucomannan (iKGM). The iKGM, now in powdered form, possesses enhanced insolubility compared to its water-soluble counterpart, making it suitable for further investigation into its prebiotic properties and effects on gut microbiota.

### 2.3. Serum Lipid Analysis

Serum triglyceride and non-esterified fatty acid (NEFA) levels were quantified using the LabAssay enzymatic chromogenic assay (Wako, Osaka, Japan) following the manufacturer’s protocols: triglycerides (https://labchem-wako.fujifilm.com/asia/product_data/docs/04572246_doc01.pdf, accessed on 7 April 2025); NEFA (https://labchem-wako.fujifilm.com/asia/product_data/docs/04572222_doc01.pdf, accessed on 7 April 2025). For triglyceride quantification, the serum sample was mixed with the enzyme reagent, and the reaction was carried out at 37 °C. The absorbance at 510 nm was measured, and triglyceride concentration was calculated based on the standard curve. For NEFA analysis, the serum was mixed with the appropriate reagents, and the reaction was performed following the manufacturer’s protocol. Absorbance at 550 nm was measured to determine NEFA concentration.

### 2.4. 16S rRNA Gene Sequencing

Fecal and small intestinal samples were collected, snap-frozen in liquid nitrogen, and stored at −80 °C. DNA extraction was performed using a modified protocol based on a previously reported method. Briefly, a small portion of mouse or human feces was suspended in 475 μL TE10 buffer (10 mM Tris-HCl, pH 8.0; 10 mM EDTA) using sterilized sticks. The suspension was incubated with lysozyme (15 mg/mL, Wako) at 37 °C for 1 h, followed by treatment with achromopeptidase (2000 U/mL, Wako) at 37 °C for 30 min. Subsequently, the sample was treated with sodium dodecyl sulfate (1% *w*/*v*) and proteinase K (1 mg/mL, Merck Ltd., Kakegawa, Japan) at 55 °C for 1 h. DNA was extracted using phenol/chloroform/isoamyl alcohol (25:24:1), precipitated with ethanol and sodium acetate, and treated with RNase A (1 mg/mL, Wako). Fragmented low-molecular-weight DNA was removed using polyethylene glycol (PEG 6000) precipitation. The V4 region of the 16S rRNA gene (515F–806R) was amplified and sequenced on an Illumina MiSeq platform using a protocol adapted from Kozich et al. [24]. PCR amplification was performed in 50 μL reactions containing 15 pmol of each primer, 0.2 mM dNTPs, 5 μL of 10× Ex Taq HS buffer, 1.25 U Ex Taq HS polymerase (Takara, Kyoto, Japan), 50 ng of extracted DNA, and sterile water. The cycling conditions included an initial denaturation at 95 °C for 2 min, followed by 25 cycles of 95 °C for 20 s, 55 °C for 15 s, and 72 °C for 1 min, with a final extension at 72 °C for 3 min. PCR products were purified using AMPure XP (Beckman Coulter, Brea, CA, USA) and quantified with a Quant-iT PicoGreen dsDNA Assay Kit (Life Technologies Japan, Osaka, Japan). Pooled libraries were analyzed on an Agilent 2100 Bioanalyzer (Agilent Technologies, San Jose, CA, USA) using the Agilent High Sensitivity DNA Kit. Quantification of pooled libraries was conducted via real-time PCR using the KAPA Library Quantification Kit for Illumina. Sequencing was performed using an Illumina MiSeq with a 500-cycle kit, generating 2 × 250 bp paired-end reads. The sequencing data were processed using QIIME (v1.8.0) and Mothur (v1.36.1).

### 2.5. Antibiotic Treatment

Mice received antibiotics in drinking water for 21 days. The antibiotic cocktail (ABX) included ampicillin (1 g/L), metronidazole (1 g/L), vancomycin (500 mg/L), and neomycin (1 g/L). A separate group received only ampicillin (1 g/L).

### 2.6. Akkermansia Muciniphila Culture

*A. muciniphila* strain BAA-835 was obtained from ATCC and cultured as per the supplier’s instructions. Bacterial cultures were centrifuged to separate supernatants and precipitates. The precipitate was resuspended in PBS to an optical density at 600 nm (OD600) of ~0.8 (4 × 10^8^ CFU). Supernatants were filtered through a 0.2 μm membrane filter (Sartorius) and diluted to match the OD600 of the precipitates before use.

### 2.7. Intestinal Metabolite Analysis

Small intestinal contents were analyzed using gas chromatography–mass spectrometry (GC/MS). Samples were collected, weighed, and mixed with a solvent solution (MeOH:H_2_O:CHCl_3_ = 2.5:1:1) containing an internal standard (2-isopropylmalic acid, 1 mg/mL; Sigma-Aldrich, Boston, MA, USA). Samples were vortexed, centrifuged at 21,000× *g* for 5 min, and the supernatant was collected. Following solvent partitioning and drying, samples were derivatized with methoxyamine hydrochloride (20 mg/mL in pyridine) and N-methyl-N-trimethylsilyl-trifluoroacetamide (MSTFA, GL Science, Tokyo, Japan). GC/MS analysis was conducted using a DB-5 capillary column (Agilent Technologies, Santa Clara, CA, USA, Tokyo, Japan) on a GCMS-TQ8030 instrument (Shimadzu, Kyoto, Japan). Data were processed using Shimadzu Smart Metabolites Database Release 3.01 and GCMS solution software Version 4.41.

### 2.8. Leptin Quantification

Leptin levels in serum samples were quantified using a commercial enzyme-linked immunosorbent assay (ELISA) kit (R&D Systems, Minneapolis, MN, USA). Briefly, serum samples were incubated with a specific antibody against leptin, followed by the addition of a substrate solution, and the absorbance was measured at [wavelength] to determine leptin concentration.

### 2.9. Statistical Analysis

Data were analyzed using GraphPad Prism 9 (GraphPad Software, Boston, MA, USA). Statistical significance was determined by Student’s *t*-test (two-tailed, unpaired) or one-way ANOVA followed by Tukey’s post hoc test. Mann-Whitney tests were also used where applicable. A *p*-value < 0.05 was considered significant (* *p* < 0.05, ** *p* < 0.01, and *** *p* < 0.001).

### 2.10. Data Availability

All datasets generated or analyzed in this study are available upon reasonable request.

## 3. Results

### 3.1. iKGM Suppresses Weight Gain

**Figure 1:**
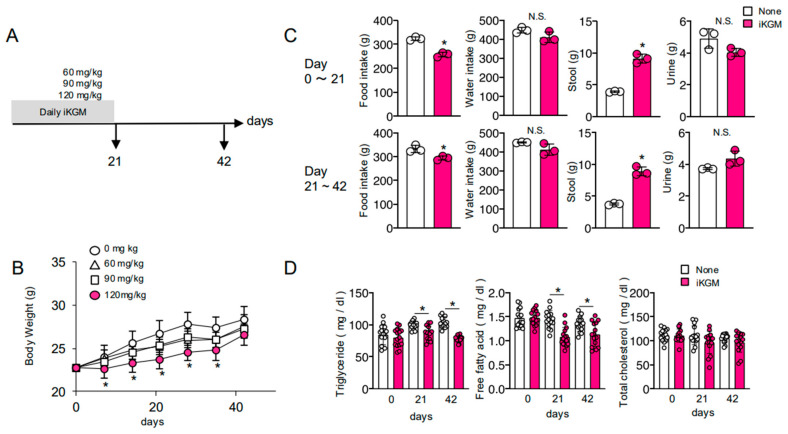
Suppression of body weight gain in mice supplemented with insoluble konjac glucomannan (iKGM). C57BL/6 mice were fed daily with various amounts of iKGM for 3 weeks through gastric intubation (**A**). Body weight of mice fed with iKGM was monitored weekly for 6 weeks. The values are mean ± SD from five mice per group. The results of one representative experiment among three repeated experiments with similar results are shown (**B**). Food and water consumption were measured weekly by subtracting the amount on day 7 from the amount on day 0 in cages containing five mice fed with 120 mg/kg iKGM. Amount of feces and urine was also measured weekly. Values indicate sum of the first (upper panels) and the next 3 weeks (lower panels) from five mice. Each dot represents results of independent individual experiments and bar graphs show mean ± SD from three experiments (**C**). Various lipid species in blood were measured at the indicated days. Each dot represents the result of individual an mouse (5 mice/experiment) in three experiments, and bar graphs show mean ± SD from 15 mice (**D**). Asterisks denote statistical significance: * *p* < 0.05 as determined by the unpaired two-tailed Student’s *t*-test. N.S. means “not significant” statistically.

To examine the effect of konnyaku-derived iKGM on body weight, iKGM was orally administered to mice fed a normal diet for 3 weeks (Figure 1A). Weight gain was significantly suppressed from the first week of administration when 120 mg/kg iKGM equivalent to 7.2 g for a 60 kg human, derived from one piece of Konnyaku, was administered (Figure 1B). While this trend persisted even with smaller doses, statistical significance was not reached (Figure 1B). Subsequently, we conducted a detailed analysis of the group receiving 120 mg/kg fiber, where a significant difference was observed. Initially, we assessed food intake and excretion, crucial factors influencing weight fluctuations. iKGM administration led to reduced food but not water intake and increased defecation volume but not urine amount (Figure 1C). Concurrently, triglycerides and free fatty acid levels in the blood remained low in the iKGM-administered group, although cholesterol levels remained unchanged (Figure 1D). These anti-obesity effects persisted for two weeks post-administration (Figure 1B). Although the amount of food intake was not measured for each individual mouse, EFR and FCR were estimated at group levels. During feeding, the iKGM until day 21 FE was 3.74 and FCR was 26.71 for the control mice, while for iKGM-treated mice it was 1.44 and 69.64, respectively. In addition to reducing food intake, poor energy efficiency was observed in mice treated with iKGM.

### 3.2. The Weight-Suppressing Effect of iKGM Depends on Intestinal Bacteria

**Figure 2:**
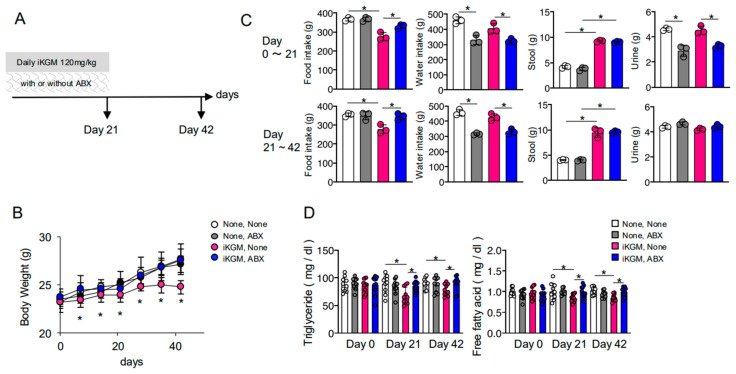
Abrogation of weight suppressing effects of iKGM by antibiotic treatment. Mice fed with 120 mg/kg iKGM were simultaneously treated with antibiotics cocktail (ABX) (**A**). Body weight of these mice were monitored weekly for 6 weeks. The values are mean ± SD from five mice per group. The results of one representative experiment among three repeated experiments with similar results are shown (**B**). Food/water input and fecal/urinal output were monitored as in Figure 1C (**C**). Triglyceride and free fatty acid were measured. Each dot represents result of an individual mouse (5 mice/experiment) in two experiments, and bar graphs show mean ± SD from 10 mice (**D**). Asterisks denotes statistical significance: * *p* < 0.05 as determined by the unpaired two-tailed Student’s *t*-test.

To explore the involvement of intestinal bacteria in iKGM’s weight-suppressing effect, we administered an antibiotic cocktail alongside iKGM (Figure 2A). Antibiotic administration alone had no effect on the body weight of mice. As previously observed, administration of iKGM significantly inhibits weight gain. However, concurrent administration of antibiotics completely nullified its suppressive effects (Figure 2B). The reduction in food intake induced by iKGM was counteracted by antibiotic treatment, although defecation volume remained increased (Figure 2C). Regardless of whether iKGM was administered or not, when antibiotics were given, the amount of water consumption decreased, which may be the cause of the decrease in urine output. This was because the bitter taste of metronidazole in the antibiotic mixture limited drinking behavior but had no effect on weight change (Figure 2C). In association with the reversal of weight suppression, the antibiotics resolved the decrease in blood lipids (Figure 2D). These findings strongly suggest that iKGM’s weight-suppressing effect is contingent upon intestinal bacteria, primarily affecting food intake rather than defecation volume.

### 3.3. iKGM Modulates the Gut Microbiota and Increases Akkermansia muciniphila

**Figure 3:**
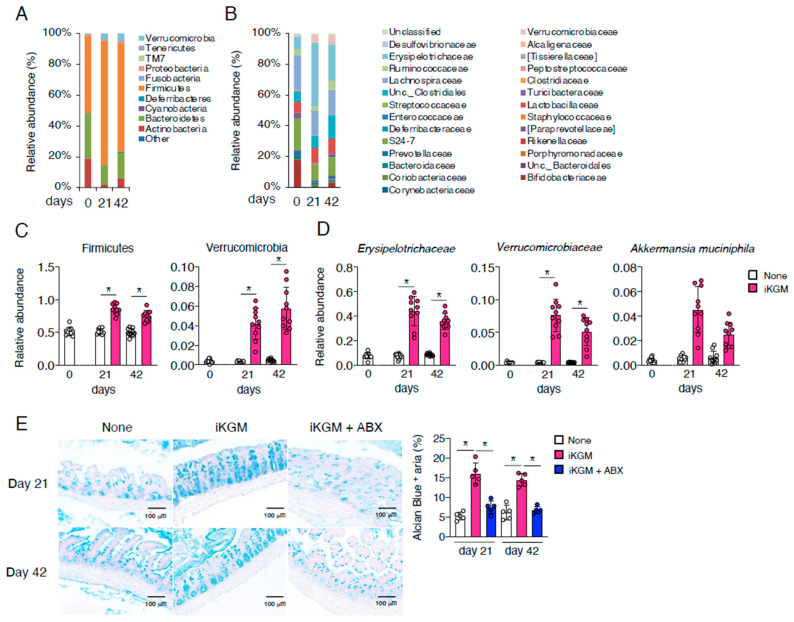
Alteration of intestinal microbiota in mice fed with iKGM. Comprehensive analyses of intestinal microbiota were performed based on 16S rRNA pyrosequencing. Relative abundance of fecal bacterial phyla (**A**), genera (**B**), Firmicutes and Verrucomicrobia (**C**), Erysipelotrichaceae, Verrucomicrobia, and *Akkermansia muciniphila* (**D**) in mice before, 21 days after, and 42 days after iKGM feeding are shown. Values in (**A**,**B**) are mean of 10 mice. In (**C**,**D**), each dot represents results of 10 individual mice, and bar graphs show mean ± SD from 10 mice. Tissues of small intestines from iKGM-treated mice on day 21 and 42 were examined to visualize mucin-producing cells by staining Alcian blue dye. Alcian blue-stained areas were digitally quantified, and each dot represents results of individual five mice and bar graphs show mean ± SD from five mice (**E**). Asterisks denote statistical significance: * *p* < 0.05 as determined by the unpaired two-tailed Student’s *t*-test.

Given the dependency of iKGM’s effects on intestinal bacteria, we conducted a comprehensive analysis of the gut microbiota. iKGM administration clearly increased Firmicutes and Verrucomicrobia at the family level (Figure 3A,C). Specifically, Erysipelotrichaceae from Firmicutes and Verrucomicrobiaceae, particularly *Akkermansia muciniphila*, exhibited notable increments at the genus and species level (Figure 3B,D). *A. muciniphila*, known for mucin degradation, also stimulates mucin production. Alcian blue staining, a technique used to indicate the presence of mucin, revealed higher mucin content in the iKGM-administered group (Figure 3E). Moreover, the antibiotic-induced depletion of intestinal bacteria abrogated the mucin-enhancing effect of iKGM (Figure 3E). These results suggest that iKGM enriches the mucin layer through modulation of intestinal microbiota, contributing to the suppression of weight gain through enhanced intestinal barrier function and altered nutrient absorption.

**Figure 4:**
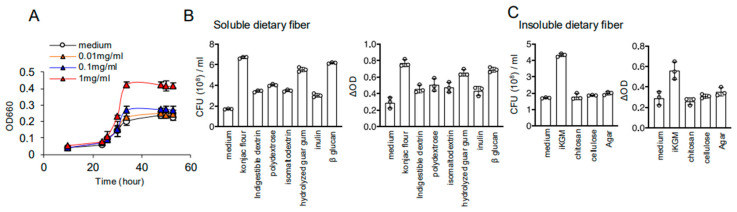
Ability of iKGM to increase *A. muciniphila. A. muciniphila* was cultured with the indicated concentration of iKGM, and turbidity of culture medium was periodically measured (**A**). Then, *A. muciniphila* was cultured with soluble (**B**) and insoluble dietary fibers (**C**) at concentration of 1 mg/mL. Turbidity of culture medium and bacterial number was determined, and each dot represents results from one independent experiment and bar graphs show mean ± SD of three repeated experiments.

We further investigated whether *A. muciniphila*, augmented in mice treated with iKGM, could proliferate in vitro. While intestinal bacteria typically assimilate water-soluble dietary fiber, in vitro studies demonstrated that water-insoluble iKGM could increase *A. muciniphila* at a concentration of 1 mg/mL (Figure 4A). At this concentration, we compared the effects of iKGM with other dietary fibers on the growth of *A. muciniphila*. Konjac flour, the raw material for iKGM, had the highest ability to increase *A. muciniphila* among water-soluble dietary fibers such as β-glucan (Figure 4B). Water-insoluble dietary fibers like agar, cellulose, and chitosan could not increase *A. muciniphila*, but only iKGM was assimilated by *A. muciniphila* and increased it (Figure 4C). Thus, this underscores iKGM’s unique ability to enhance *A. muciniphila* growth even in vitro.

### 3.4. Alteration of Intestinal Bacteria by iKGM Increases SCFAs and Leptin

**Figure 5:**
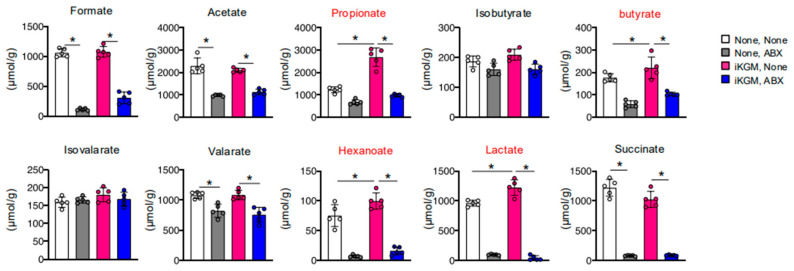
Impact of iKGM treatment on intestinal metabolites. Several metabolites, specifically, water-soluble acids including SCFA in intestinal contents obtained from mice 21 days after iKGM treatment were quantified. Each dot represents a result from an individual mouse, and bar graphs show the mean ± SD of five mice. The results of one representative experiment among two repeated experiments with similar results are shown. Compounds shown in red are significantly changed after iKGM administration. Asterisks denote statistical significance: * *p* < 0.05 as determined by the unpaired two-tailed Student’s *t*-test.

**Figure 6:**
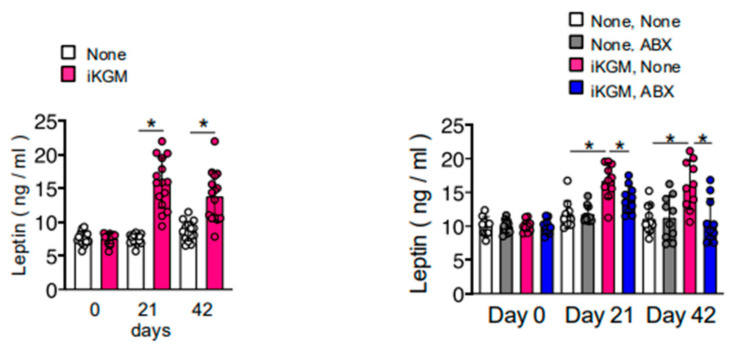
Increased serum leptin concentration in mice treated with iKGM dependent on intestinal microbiota. Serum samples obtained from the indicated mice were used for leptin quantification. Each dot represents a result from an individual 10 mice, and bar graphs show mean ± SD of 10 mice. The results of one representative experiment among two repeated experiments with similar results are shown. Asterisks denote statistical significance: * *p* < 0.05 as determined by the unpaired two-tailed Student’s *t*-test.

We explored the effects of iKGM-mediated modulation of intestinal bacteria on intestinal metabolites, as an increase in mucin can influence the growth of gut microbiota, potentially altering the gut flora and promoting the production of SCFAs, which are known to play a role in metabolic regulation. Administration of iKGM led to an increase in SCFAs, such as propionate, butyrate, and hexanoate, along with lactate (Figure 5). The increase in all these metabolites was abolished by antibiotic administration (Figure 5). SCFAs have many physiological functions related to weight control, including the induction of the secretion of leptin, a potent appetite suppressor. As the weight-suppressing effects of iKGM seem to be associated with a decrease in food intake, we measured blood leptin concentrations in mice treated with iKGM. It exhibited elevated levels with iKGM administration, which were restored upon antibiotic treatment (Figure 6). Taken together, these findings suggest that iKGM influences intestinal bacteria to produce SCFAs, subsequently triggering leptin secretion and altering feeding behavior, ultimately suppressing weight gain.

## 4. Discussion

In this study, we investigated the weight-suppressing effects of insoluble dietary fiber derived from “konnyaku”, a traditional Japanese food made from the tuber of *Amorphophallus konjac*. This is the first report to examine the effects of insoluble konjac glucomannan (iKGM) on weight suppression, complementing earlier studies that primarily focused on the water-soluble form of KGM [21,22,23]. The results from this study contribute to the growing body of evidence suggesting that konjac-derived fibers can influence body weight regulation and metabolic health.

The weight-suppressing effect of iKGM appears to be mainly due to a decrease in food intake and low energy efficiency, both of which are mediated by alterations in the gut microbiota. In addition, iKGM also increases fecal volume, likely due to its water-binding properties and bulk-forming effect, which is typical of insoluble fibers, though less related to weight control. Previous research has shown that insoluble fibers can increase mucin production [25], and this was corroborated in our study. iKGM increases mucin production in the small intestines through prebiotic characteristics, including the enhancement of *A. muciniphila*. The thickened mucin layer in the intestines can act as a physical barrier, reducing the uptake of nutrients. Low energy efficiency (EFR) was thought to be due to increased calorie expenditure and decreased absorption of nutrients. In considering that the mucous membrane became thicker, the latter is thought to have contributed more. The thickened mucin layer in the intestines also dampens the uptake of potentially harmful substances such as lipopolysaccharides (LPS), a bacterial molecular pattern that induces inflammation [26]. Since microinflammation in adipose tissue is also involved in obesity [27,28,29], reducing LPS uptake may further enhance the anti-obesity effects of iKGM.

The increase in mucin production likely contributes to enhanced gut barrier function, leading to suppression of nutrient uptake. On the other hand, mucin composed of polysaccharides can serve as a substrate for certain gut bacteria, such as those from the *Erysipelotrichaceae* family, which are known to break down mucin and proliferate. This dynamic interaction between mucin production and gut microbiota composition warrants further exploration to fully understand its implications for weight control.

The impact of iKGM on the production of short-chain fatty acids (SCFAs) is another important mechanism through which it exerts its effects. SCFAs, including butyrate, propionate, and acetate, are known to play a critical role in regulating energy metabolism. Specifically, *A. muciniphila* has been shown to produce propionate through the fermentation of fucose, a sugar abundant in mucin [30,31]. The increase in propionate observed in our study may therefore be attributed to the activity of *A. muciniphila*, although the bacteria responsible for lactate and butyrate production in this context remain unidentified. SCFAs have been extensively studied for their role in appetite regulation, energy balance, and fat metabolism. They induce leptin secretion from adipocytes [32], contributing to the suppression of appetite and subsequent weight reduction. Furthermore, SCFAs also act on intestinal endocrine cells to stimulate the release of hormones like peptide YY and glucagon-like peptide-1, which control food intake and regulate sugar and lipid metabolism [33,34]. Additionally, SCFAs can stimulate the sympathetic nervous system to increase energy expenditure by promoting the release of noradrenaline [35]. These mechanisms highlight the multifaceted role of SCFAs in energy homeostasis and underscore the potential of iKGM as an effective prebiotic agent for obesity management.

While this study provides valuable insights into the mechanisms through which iKGM influences body weight, it is important to consider the broader context of dietary fiber and its role in weight management. For example, a recent study by Smith et al. [36] found that other forms of dietary fiber, such as those derived from fruits and vegetables, can also modulate gut microbiota and promote weight loss by increasing SCFA production and improving metabolic health. Furthermore, fiber intake, particularly soluble fiber, is associated with lower risks of obesity and metabolic diseases, suggesting that a balanced intake of both soluble and insoluble fibers may be optimal for weight control [37]. In addition to its effects on gut microbiota, konnyaku consumption has been associated with lower rates of obesity in Japan [38], where it is widely consumed. This observation highlights the potential of konnyaku-derived dietary fibers to contribute to weight management in diverse populations, further supporting the relevance of this study’s findings to global health [39].

However, there are several limitations to this study. One of the major limitations is that the experiments were conducted using animal models, and thus, the findings may not fully reflect human responses. Additionally, the long-term effects of iKGM on metabolic health were not evaluated, and further research is needed to examine its sustained impact. Moreover, although we observed increased propionate production, the specific bacteria responsible for the production of lactate and butyrate remain unidentified, and future studies should aim to identify these species to gain a more comprehensive understanding of iKGM’s impact on gut microbiota.

Future perspectives include the need for clinical trials in humans to validate the effectiveness of iKGM as a therapeutic agent for obesity prevention and management. Furthermore, long-term studies are needed to assess the sustained impact of iKGM on metabolic health. Additionally, future research should focus on investigating the dynamics between mucin production, gut microbiota, and the various metabolic pathways involved in weight regulation to explore further the therapeutic potential of iKGM in obesity management.

## Data Availability

The raw data supporting the conclusions of this article will be made available by the authors on request.

## Abbreviations

KF: konjac flour; iKGM: insoluble glucomannan; SCFAs: short-chain fatty acids; ABX: combination of antibiotics; GC/MS: gas chromatography/mass spectrometry; OD: optical density; CFU: colony-forming unit; MRM: multiple reaction monitoring.
